# Supplementary material for: HTS and PCR Methods Are the Most Used in the Diagnosis of Aspergillosis: Advantages over Other Molecular Methods
Source: J Fungi (Basel). 2025 Oct 6;11(10):720. doi: 10.3390/jof11100720 (PMC12565454; doi:10.3390/jof11100720)
Supplement: Supplementary file 1 [file jof-11-00720-s001.zip › table 1.pdf]

Table S1. Molecular techniques used for the diagnosis of aspergillosis with clinical samples.

NGS				
**Species identified**	**Clinical presentation**	**Clinical sample**	**Sensitivity, specificity, accuracy or detection rate (%)**	**Refere nce**
Aspergillus oryzae	CNS aspergillosis	CSF	ND	[44]
Aspergillus	Patients with lung transplantation	BALF, TBLB	ND	[45]
A. versicolor<br>*A. flavus*	Bell's palsy	Tissue	ND	[46]
A. fumigatus	PA	BALF	ND	[47]
A. fumigatus	CNS aspergillosis	CSF and BALF	ND	[48]
Aspergillus	PA	BALF	Sensitivity 100<br>Specificity 93.3	[49]
Aspergillus spp.	PA	BALF, blood and pleural fluid	Sensitivity 91.7<br>Specificity 71.4	[50]
Aspergillus spp.	IPA	Blood, BALF, and sputum	ND	[51]
A. niger	CNS aspergillosis	CSF	ND	[52]
Aspergillus spp.<br>*A. fumigatus*<br>*A. flavus*<br>*A. terrestris*<br>*A. oryzae*	IPA	BALF	Sensitivity 82.6<br>Specificity 97.7<br>Accuracy 92.5	[53]
A. flavus	Malignant external otitis	Tissue	ND	[54]
A. flavus	PA	BALF	ND	[55]
Aspergillus	IPA	BALF	**Accuracy**:<br>Whole population 80.5<br>Immunocompromised patients 73.7<br>Diabetic patients 85.3	[56]
Aspergillus spp.	IPA	Blood and BALF	ND	[57]
A. fumigatus	Mediastinal *Aspergillus fumigatus* abscess	Transbronchial needle aspiration	ND	[58]
Aspergillus spp.	IPA	BALF	**GM:**<br>Sensitivity 57.7<br>**mNGS:**<br>Sensitivity 42.3<br>**Culture**:<br>Sensitivity 30.8<br>**Smear**:<br>Sensitivity 7.7	[59]
A. lentulus<br>*A. fumigatus*	CAPA	Plasma	Sensitivity 83<br>Specificity 97	[60]
A. lentulus	PA	BALF	ND	[61]
Aspergillus spp.	CNS aspergillosis	CSF	ND	[62]

A. fumigatus<br>*A. flavus*<br>*A. oryzae*	IPA	BALF	ND	[63]
A. flavus	ALL	Blood, CSF, and BALF	**Detection rate**:<br>mNGS 57.2<br>Culture 12.5	[64]
A. fumigatus	PA	BALF	**Detection rate**:<br>BALF-mNGS (cfDNA, wcDNA) 57.2<br>Conventional microbiological tests 12.5	[65]
Aspergillus spp.	IPA	BALF	Combining fungal culture with the GM test and mNGS<br>Sensitivity 80.6<br>Specificity 92.2	[66]
A. sydowii	PA	BALF	ND	[67]
Aspergillus	Infectious keratitis	Tissue	ND	[68]
Aspergillus spp.	PA	BALF	ROC curve result for BALF mNGS indicated an area under the curve of 0.894 (95%CI: 0.811-0.976), with an optimal threshold value of 23 for discriminating between *Aspergillus* infection and colonization	[69]
A. fumigatus.	Bronchiectasis	BALF, sputum, and blood	**Detection rate**:<br>mNGS 98.5<br>BALF culture 28.4<br>Sputum culture 17.5<br>Blood culture 0	[70]
Aspergillus	PA	BALF	**Detection rate:**<br>*Aspergillus* 8.9	[71]
A. fumigatus	Endocarditis	Blood	ND	[72]
A. flavus<br>*A. oryzae*<br>*A. fumigatus*	COVID-19 and ARDS	BALF, and blood	ND	[73]
**NTS**				
A. fumigatus	CNS aspergillosis	CSF	ND	[74]
A. flavus	IPA	Blood	ND	[75]
**ISH**				
Aspergillus sp.	ND	Tissue	ND	[76]
Aspergillus spp.	AFS	ND	ND	[77]
Aspergilosis spp.	IPA	Tissue	Specificity 100	[78]
Aspergillus spp.	Patients with hematologic malignancies	Tissue	Detection rate 92.3	[79]
Aspergillus spp.	AIAR	Tissue	ND	[80]

Aspergillus spp.	IMSA	Tissue	ND	[81]
A. fumigatus	IFI	Tissue	ND	[82]
A. fumigatus	ND	Sputum, BALF	Sensitivity 79<br>Specificity 100	[83]
**MICROARRAYS**				
A. fumigatus<br>*A. flavus*<br>*A. niger*<br>*A. terreus*	ND	ND		[84]
A. fumigatus<br>*A. flavus*<br>*A. terreus*	Neutropenic patients	Blood, BALF, and tissue	ND	[85]
Aspergillus allergen	Patients with severe asthma	Blood	ND	[86]
**PCR-RFLP**				
Aspergillus spp.	Neutropenic patients	Blood	ND	[87]
**LAMP**				
A. fumigatus	IA	BALF and blood	Sensitivity: 89.19<br>Specificity: 53.13	[88]
A. fumigatus	ND	ND	ND	[89]
A. fumigatus	CPA	Sputum	Sensitivity: 55.9<br>Specificity: 100	[90]
A. fumigatus	IA	Sputum	Specificity: 100	[91]

NGS: Metagenomic Next Generation Sequencing; **NTS**: Nanopore Targeted Sequencing**;** **CNS**: central nervous system; **CSF**: cerebrospinal fluid; **ND**: Non determined; **BALF**: Bronchoalveolar lavage fluid; **TBLB**: transbronchial lung biopsy; **PA**: Pulmonary aspergillosis; **IPA**: Invasive pulmonary aspergilosis; **GM**: Galactomannan; **CAPA**: COVID-19-associated pulmonary aspergillosis; **ALL**: Acute lymphoblastic leukemia; **cfDNA**: Cell-free DNA; **wcDNA**: Whole-cell DNA; **ROC**: Receiver operating characteristic; **ARDS**: Acute respiratory distress syndrome; **ISH**: In situ hybridization; **AFS**: Allergic fungal sinusitis; **AIAR**: Acute invasive aspergillus rhinosinusitis; **IMSA**: Invasive maxillary sinus aspergillosis; **IFI**: Invasive fungal infections; **RFLP**: Restriction fragment length polymorphism; **LAMP**: Loop-Mediated Isothermal Amplification; **IA**: Invasive aspergillosis; **CPA**: Chronic Pulmonary Aspergillosis
