# Supplementary material for: HTS and PCR Methods Are the Most Used in the Diagnosis of Aspergillosis: Advantages over Other Molecular Methods
Source: J Fungi (Basel). 2025 Oct 6;11(10):720. doi: 10.3390/jof11100720 (PMC12565454; doi:10.3390/jof11100720)
Supplement: Supplementary file 1 [file jof-11-00720-s001.zip › table 2.pdf]

Table S2. The PCR method for the diagnosis of aspergillosis using clinical samples**.**

**Specie**	**Clinical form**	**Target**	**Clinical sample**	**Sensitivity and specificity (%)**	**Referenc e**
**qPCR**					
A. fumigatus	IA	18S rRNA	Serum	Sensitivity: 43.8<br>Specificity: 63.2	[92]
A. fumigatus<br>*A. terreus*<br>*A. niger*<br>*A. flavus*	IA	28S rRNA	Plasma<br>Serum<br>Blood	Sensitivity: 85.75<br>Specificity: 87.5	[93]
A. fumigatus	IA	Orthologous genes of facC	BALF	NA	[94]
Aspergillus spp.	IA	*ITS1* and 5.8S rRNA	Plasma<br>Serum	Serum<br>Sensitivity: 68.4<br>Specificity: 76.2<br>Plasma<br>Sensitivity: 94.7<br>Specificity: 83.3	[95]
A. fumigatus	IA	18S rRNA	Serum	Specificity: 84.3	[96]
Aspergillus spp	IA	NA	Serum	Sensitivity: 90%<br>Specificity: 73.3	[97]
Aspergillus spp.	IA	*ITS1*/5.8S rRNA	Blood	Sensitivity: 100<br>Specificity: 63	[98]
Aspergillus spp.	IA	*ITS1*<br>5.8S rRNA	Plasma<br>Serum<br>Blood	Plasma<br>Sensitivity: 91<br>Specificity: 53<br>Serum<br>Sensitivity: 80<br>Specificity: 69<br>Blood<br>Sensitivity: 55<br>Specificity: 96	[99]
A. fumigatus	IA	28S rRNA and TR_{34}/L98H mutation	BALF<br>Serum	BALF<br>Sensitivity: 92.9<br>Specificity: 90.1<br>Serum<br>Sensitivity: 100<br>Specificity: 84.6	[100]
Aspergillus spp.	IA	28S rRNA	BALF	Sensitivity: 90<br>Specificity: 97	[101]

A. fumigatus	Fungal rhinosinusitis	NA	Fungus balls	NA	[102]
Aspergillus spp.	IA	18S rRNA	Blood	NA	[103]
Aspergillus spp.	IA	28S rRNA	BALF	Supernatant (BALF)<br>Sensitivity: 57.1<br>Specificity: 100<br>Pellet (BALF)<br>Sensitivity: 50.0<br>Specificity: 100	[104]
A. fumigatus<br>*A. niger*	CAPA	28S rRNA	Serum<br>BALF	NA	[105]
Aspergillus spp.	IA	18S rRNA and 28S rRNA	Serum	NA	[106]
Aspergillus spp.	CPA	18S rRNA	BALF	Sensitivity: 87.18<br>Specificity: 89.80	[107]
Aspergillus spp.	IA	28S rRNA	Serum	Sensitivity: 62<br>Specificity: 33	[108]
A. fumigatus<br>*A. flavus*<br>*A. niger*	IA	NA	BALF<br>Sputum<br>Tissue<br>Serum	NA	[109]
Aspergillus spp.	PA	NA	BALF	Sensitivity: 82.4<br>Specificity: 98.3	[110]
Aspergillus spp.	IA	18S rRNA, 28S rRNA, 5.8S rRNA and *ITS*	Sputum<br>BALF<br>Endotracheal aspirate<br>Tissue	Sensitivity: 92.5<br>Specificity: 81.4	[111]
Aspergillus spp	IA	18S rRNA	BALF	Sensitivity: 100<br>Specificity: 87	[112]
Aspergillus spp.	IA	NA	BALF	Sensitivity: 90<br>Specificity: 92.5	[113]
A. fumigatus	IA	28S rRNA	BALF	Sensitivity: 40<br>Specificity: 69	[114]

A. flavus<br>*A. fumigatus*<br>*A. niger*<br>*A. nidulans*<br>*A. sydowii*<br>*A. lentulus*<br>*A. glaucus*	IA	18S rRNA, 28S rRNA 5S and *ITS*	BALF<br>Tissue	Sensitivity: 93.3<br>Specificity: 97.1	[115]
A. fumigatus	IA	18S rRNA	BALF	Sensitivity: 50	[116]
Aspergillus spp.	IA	NA	BALF	Sensitivity: 94.1<br>Specificity: 76.5	[117]
A. fumigatus	IA	NA	BALF<br>Endotracheal aspirate<br>Pleural fluid<br>Pericardial fluid	NA	[118]
Aspergillus spp	CAPA	28S rRNA	BALF<br>Sputum<br>Endotracheal aspirate	Sensitivity: 88.9<br>Specificity: 57.1	[119]
**Multiplex PCR**					
A. fumigatus	IA	28S rRNA	Serum	Sensitivity: 78.6<br>Specificity: 100	[120]
Aspergillus spp.<br>*A. fumigatus*<br>*A. terreus*	IA	28S rRNA	BALF	Sensitivity: 84<br>Specificity: 80	[121]
Aspergillus spp<br>*A. fumigatus*<br>*A. terreus*	IA	28S rRNA	Plasma	Sensitivity: 80<br>Specificity: 77.8	[122]
A. fumigatus	IA	28S rRNA	BALF<br>CSF	NA	[123]
Aspergillus spp.	IA	28S rRNA	BALF<br>Blood	NA	[124]
Aspergillus spp.	IA	18S rRNA	Serum	Arthus PCR<br>Sensitivity: 47.6<br>Specificity: 100<br>MycAssay<br>Sensitivity: 61.9<br>Specificity: 100	[125]
Aspergillus spp.	CAPA	28S rRNA	Endotracheal aspirate	NA	[126]
A. fumigatus	IA	28S rRNA	BALF	Sensitivity: 79.3<br>Specificity: 90	[127]

A. fumigatus	IA	28S rRNA	BAF	Sensitivity: 52.0<br>Specificity: 64.7	[128]
Aspergillus spp.	IA	28S rRNA	BALF	Sensitivity: 0.71<br>Specificity: 0.73	[129]
A. fumigatus<br>*A. flavus*<br>*A. niger*<br>*A. nidulans*<br>*A. terreus*<br>*A. versicolor*	IA	Genes SCW4	BALF	NA	[130]
A. fumigatus<br>*A. flavus*<br>*A. terreus*<br>*A. niger*	Aspergillosis	*ITS*	Tissue	NA	[131]
**Nested PCR**					
Aspergillus spp.	IA	18S rRNA	BALF	Sensitivity: 70<br>Specificity: 100	[132]
Aspergillus spp.	IA	18S rRNA	BALF	Sensitivity: 32<br>Specificity: 100	[133]
Aspergillus spp.	IA	28S rRNA	BALF<br>Blood	BALF<br>Sensitivity: 44<br>Blood<br>Sensitivity: 0	[134]
Aspergillus spp.	CPA	28S rRNA	BALF	Sensitivity: 86.7 and 66.7<br>Specificity: 84.2 and 94.2	[135]
Aspergillus spp	IA	NA	BALF	Sensitivity: 91<br>Specificity: 97	[136]
Aspergillus spp.	IA	18S rRNA	BALF<br>Serum	BALF<br>Sensitivity: 44<br>Specificity: 94<br>Serum<br>Sensitivity: 11<br>Specificity: 97	[137]
Aspergillus spp.	Sinusitis crónica (rinosinusitis)	18S rRNA	Tissue	Sensitivity: 90<br>Specificity: 98.3	[138]
Aspergillus spp.	IA	18S rRNA	BALF	BALF	[139]

			CSF<br>Tissue<br>Blood	Sensitivity: 64<br>Specificity: 85<br>CSF<br>Sensitivity: 100<br>Specificity: 85<br>Tissue<br>Sensitivity: 67<br>Specificity: 85<br>Blood<br>Sensitivity: 8<br>Specificity: 85	
Aspergillus spp.	IA	18S rRNA	BALF	Sensitivity: 40<br>Specificity: 93	[140]
Aspergillus spp.	IA	18S rRNA	BALF	Sensitivity: 80<br>Specificity: 74	[141]
Aspergillus spp.	IA	18S rRNA	BALF	Sensitivity: 77.8<br>Specificity: 78.6	[142]
**RT-PCR**					
A. fumigatus	IA	18S rRNA	Blood	Sensitivity: 72.22<br>Specificity: 84	[143]
**PCR**					
A. citrinoterreus	IA	*BenA*<br>*CaM*<br>*ITS*	Sputum<br>Bronchial secretion<br>Wound<br>Abscess	NA	[144]
A. fumigatus<br>*A. flavus*<br>*A. niger*<br>*A. terreus*	IA	18S rRNA	BALF<br>Pleural fluid<br>CSF<br>Synovial fluid	NA	[145]
**Universal Digital High-Resolution Melt (U-dHRM)**					
A. fumigatus<br>*A. flavus*<br>*A. terreus*<br>*A. nidulans*<br>*A. niger versicolor*	IPA		BALF	ND	[26]
A. fumigatus<br>*A. flavus*	IPA		Plasma<br>BALF	Sensitivity 73.1%<br>Specificity 94.4%	[27]

A. niger<br><br>*A. terreus*					

qPCR: Real-time PCR; **IA**: Invasive aspergillosis; **BALF**: Bronchoalveolar lavage fluid; **CAPA**: COVID-19-associated pulmonary aspergillosis; **CPA**: Chronic pulmonary aspergillosis; **PA**: Pulmonary aspergillosis; **CSF**: Cerebrospinal fluid.
